# Efficient Degradation of Tetracycline Antibiotics by Engineered Myoglobin with High Peroxidase Activity

**DOI:** 10.3390/molecules27248660

**Published:** 2022-12-07

**Authors:** Guang-Rong Wu, Li-Juan Sun, Jia-Kun Xu, Shu-Qin Gao, Xiang-Shi Tan, Ying-Wu Lin

**Affiliations:** 1 School of Chemistry and Chemical Engineering, University of South China, Hengyang 421001, China; 2 Hengyang Medical School, University of South China, Hengyang 421001, China; 3 Key Laboratory of Sustainable Development of Polar Fisheries, Yellow Sea Fisheries Research Institute, Chinese Academy of Fishery Sciences, Qingdao 266071, China; 4 Department of Chemistry & Institute of Biomedical Science, Fudan University, Shanghai 200433, China

**Keywords:** heme enzyme, myoglobin, antibiotics, degradation, environmental remediation

## Abstract

Tetracyclines are one class of widely used antibiotics. Meanwhile, due to abuse and improper disposal, they are often detected in wastewater, which causes a series of environmental problems and poses a threat to human health and safety. As an efficient and environmentally friendly method, enzymatic catalysis has attracted much attention. In previous studies, we have designed an efficient peroxidase (F43Y/P88W/F138W Mb, termed YWW Mb) based on the protein scaffold of myoglobin (Mb), an O_2_ carrier, by modifying the heme active center and introducing two Trp residues. In this study, we further applied it to degrade the tetracycline antibiotics. Both UV-Vis and HPLC studies showed that the triple mutant YWW Mb was able to catalyze the degradation of tetracycline, oxytetracycline, doxycycline, and chlortetracycline effectively, with a degradation rate of ~100%, ~98%, ~94%, and ~90%, respectively, within 5 min by using H_2_O_2_ as an oxidant. These activities are much higher than those of wild-type Mb and other heme enzymes such as manganese peroxidase. As further analyzed by UPLC-ESI-MS, we identified multiple degradation products and thus proposed possible degradation mechanisms. In addition, the toxicity of the products was analyzed by using in vitro antibacterial experiments of *E. coli*. Therefore, this study indicates that the engineered heme enzyme has potential applications for environmental remediation by degradation of tetracycline antibiotics.

## 1. Introduction

**Figure 1:**
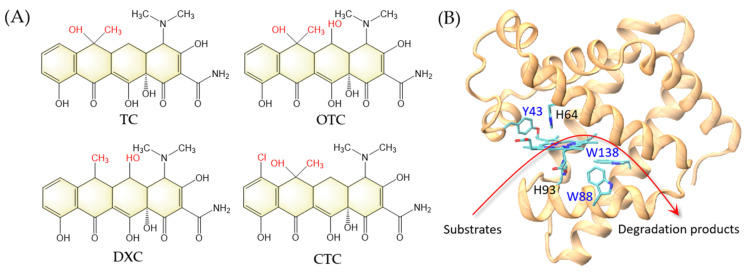
(**A**) Chemical structures of tetracycline (TC), oxytetracycline (OTC), doxycycline (DXC), and chlortetracycline (CTC). The structural differences are highlighted in red. (**B**) A model structure of F43Y/P88W/F138W Mb based on the X-ray structure of F43Y Mb (PDB code 4QAU [8]). The arrow indicates the peroxidase activity by degradation of substrates.

With the rapid development of global society, water pollution causes serious public health problems. The widely-used antibiotics remaining in water are considered to be an emerging class of water pollutants because of their long-term cumulative negative effects on the ecosystems [1]. For example, tetracycline antibiotics are a broad-spectrum class of antibiotics, including tetracycline (TC), oxytetracycline (OTC), chlortetracycline (CTC), and doxycycline (DXC) (Figure 1A), which have been widely used in the pharmaceutical industry and animal husbandry [2,3]. Meanwhile, due to abuse and improper disposal, they are ubiquitous in aquatic environments [4]. In addition to causing chemical pollution, antibiotics in the environment may induce the generation of resistance genes in microorganisms. [5,6]. These drug-resistant microorganisms may enter the human body through direct or indirect ways, leading to the enhancement of drug resistance in the human body, which may pose a threat to human health and safety [7]. Therefore, it is urgent to effectively remove antibiotics from the wastewater and protect the ecological environment.

To date, many methods have been developed to remove antibiotics from the aquatic environment, and the most commonly used methods are adsorption [9,10], chemical [11,12], and biological methods [13,14]. Although the adsorption treatment of pollutants will not produce toxic substances, the pollutants cannot be fundamentally removed and they may also cause secondary pollution [15]. The chemical process can treat highly concentrated antibiotics in wastewater, but additional chemical reagents are required to be added, which greatly increases the cost of wastewater treatment. Therefore, this process has not been widely used to remove pollutants from wastewater [16]. Comparatively, biological methods have several advantages, such as simple operation in mild reaction conditions, without secondary pollution, and being environmentally friendly [17,18]. These advantages make biological methods have great applications in the removal of antibiotics from wastewater.

In recent years, the use of enzymes in organisms to catalyze the degradation of emerging pollutants in the environment is a hot spot in biological methods, which has attracted extensive attention for many researchers. Recently, as found in eukaryotes and prokaryotes, the heme-containing peroxidase uses H_2_O_2_ as an oxidant to catalyze the oxidation of a variety of substrates including reactive blue dyes, 2,4,6-trichlorophenol, antibiotics, etc. [18,19,20]. Moreover, other metalloenzymes such as laccase, manganese peroxidase (MnP), horseradish peroxidase (HRP), and metallo-β-lactamase (MBL) can also play a catalytic role in the biodegradation of antibiotics [14,17,21,22].

In previous studies, we have designed several efficient peroxidases based on the protein scaffold of myoglobin (Mb), an O_2_ carrier, by modifying the heme center. For example, by introducing a Tyr residue in the heme center, we identified a novel post-translational modification in the F43Y Mb mutant that forms a novel Tyr-heme crosslinking and exhibits enhanced peroxidase activity [8]. Moreover, inspired by the structural feature of native dye-decolorizing peroxidase (DyPs) that contain multiple Trp residues [23,24], we constructed a triple mutant F43Y/F138W/P88W Mb (named YWW Mb) by further introducing two Trp residues around the heme center in F43Y Mb (Figure 1B). We showed that the triple mutant is efficient in both dye decolorization and lignin bioconversion [25,26].

In this study, we have achieved satisfactory results in the application of the triple mutant for the degradation of tetracycline antibiotics in the aqueous environment. It showed that the triple mutant with high peroxidase activity could catalyze the degradation of tetracycline antibiotics efficiently, producing non-toxic degradation products, which suggests potential applications for environmental remediation.

## 2. Results and Discussion

### 2.1. UV-Vis Studies

**Figure 2:**
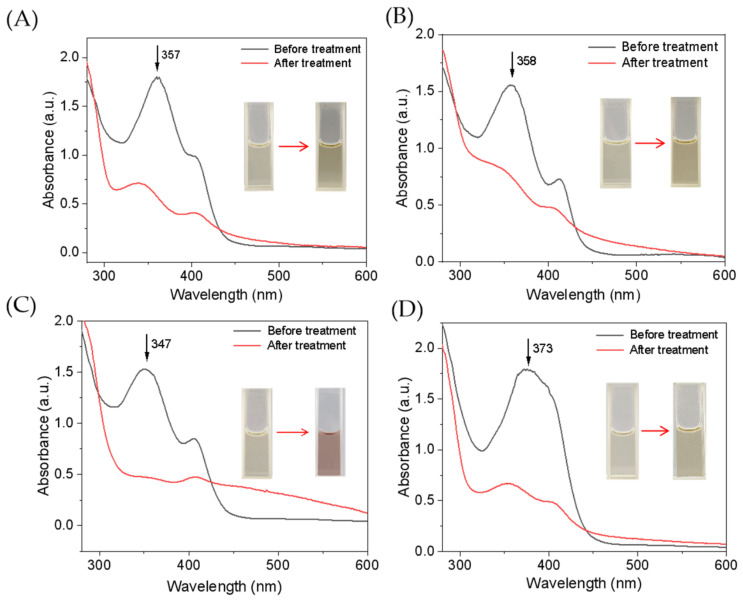
UV–vis absorption spectra of tetracycline antibiotics (100 µM) before and after treatment by F43Y/P88W/F138W Mb (5 µM) in the presence of H_2_O_2_ (400 µM) for 5 min: (**A**) TC, (**B**) OTC, (**C**) DXC, and (**D**) CTC. Inset shows the color change of the substrate solution before and after degradation.

The single mutant F43Y Mb and triple mutant YWW Mb were expressed and purified as reported previously [25]. We first evaluated the catalytic activity of YWW Mb in the oxidation of different tetracycline antibiotics, including tetracycline (TC), oxytetracycline (OTC), doxycycline (DXC), and chlortetracycline (CTC). All experiments were carried out on a UV-vis spectrophotometer under optimal reaction conditions (i.e., pH 7.0, 25 mM potassium phosphate buffer, with an addition of 0.4 mM H_2_O_2_). The spectral changes of these antibiotics upon oxidation were monitored after incubation for 5–30 min. As shown in Figure 2, the enzyme YWW Mb was capable to degrade all the substrates tested, with large decreases in the characteristic absorptions and marked color changes. Particularly, it was observed that the absorbance of DXC at 347 nm almost decreased completely after incubation for 5 min (Figure 2C), indicating efficient degradation. Moreover, the Soret band remained at around 408 nm, which indicates the engineered enzyme was stable in the catalytic reaction. Therefore, it would be meaningful to further investigate the degradation activity of Mbs and explore the mechanism for the four antibiotics.

### 2.2. Kinetic Studies

**Figure 3:**
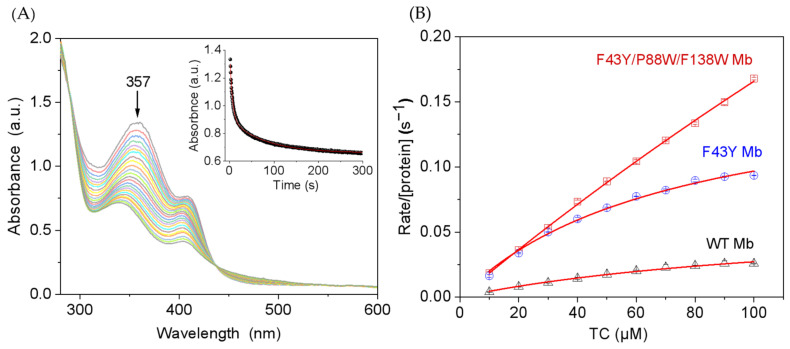
(**A**) Kinetic study of TC (100 μM) oxidation in the presence of H_2_O_2_ (0.4 mM) catalyzed by F43Y/P88W/F138W Mb (5 μM). Time-dependent change of the absorbance at 357 nm was shown as inset and was fitted to the double-exponential decay function. (**B**) Steady-state rates of H_2_O_2_-dependent oxidation of TC catalyzed by F43Y/F138W/P88W Mb, F43Y Mb, and WT Mb, with increasing substrate concentrations. Reaction conditions: 5 μM protein, 400 μM H_2_O_2_, 25 mM potassium phosphate buffer, pH 7.0, 25 °C. The data were fitted to the Michaelis-Menten equation. The error bar was generated by three triplicate experiments.

Considering the structural similarities of tetracycline antibiotics, we chose TC as a representative substrate and carried out kinetic studies for its degradation-catalyzed YWW Mb. The degradation of TC was monitored by the absorbance change at 357 nm. As shown in Figure 3A for the catalysis by YWW Mb, the absorbance decreased rapidly for the first ~25 s, with an apparent rate constant (*k_obs_*) of 0.182 ± 0.008 s^−1^. Moreover, we studied the dependence of TC concentrations on the enzymatic activity of YWW Mb, with those of F43Y Mb and wild-type (WT Mb) as controls. As shown in Figure 3B, the obtained catalytic parameters showed that YWW Mb exhibited a high catalytic rate (*k*_cat_ = 144 ± 24 s^−1^), which is ~9-fold and 23-fold higher than that of F43Y Mb (*k*_cat_ = 16.8 ± 0.92 s^−1^) and WT Mb (*k*_cat_ = 6.2 ± 0.4 s^−1^), respectively. These observations indicated that with two additional Trp residues introduced close to the heme active site, the triple mutant protein YWW Mb exhibits the highest catalytic activity in the degradation of TC.

### 2.3. HPLC Studies

**Figure 4:**
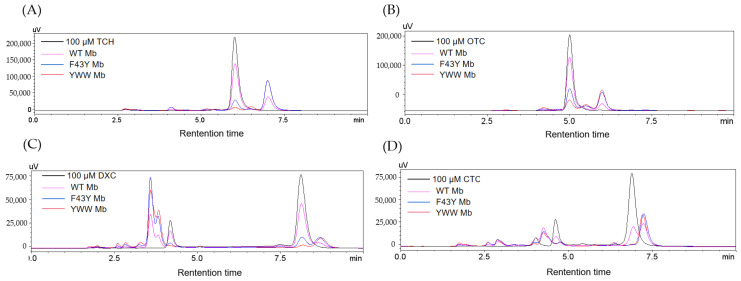
HPLC elution profiles monitored at 280 nm of the reaction solution containing (**A**) TC, (**B**) OTC, (**C**) DXC, (**D**) CTC (100 µM), respectively, after treatment with WT Mb and its mutants (5 µM) in the presence of H_2_O_2_ (400 μM) for 5 min.

To further confirm the degradation of the antibiotics of TC, OTC, DXC, and CTC catalyzed by the Mb mutants, we performed an HPLC analysis of the reaction solutions. As shown in Figure 4 for the HPLC elution profiles monitored at 280 nm of the reaction solution containing each antibiotic, as treated with YWW Mb, F43Y Mb, or WT Mb for 5 min in the presence of 400 μM H_2_O_2_. From the changes in signal intensity of the four antibiotics (with a retention time of 6.005, 5.004, 8.121, and 6.890 min for TC, OTC, DXC, and CTC, respectively), YWW Mb exhibited the most efficient degradation activity toward the substrates, followed by F43Y Mb. Comparatively, WT Mb showed the lowest activity in the degradation of the four substrates. Moreover, the degradation products of TC and OTC were observed for the retention time of 6~7 min. Furthermore, some intermediate products were formed in the solutions of DXC and CTC upon degradation. As shown in Figure 4C for DXC degradation, conspicuous peaks were observed in both 2~5 and 8.5~9 min, which indicates that multiple intermediate compounds were produced during the degradation process. Similar situations were also found in the degradation of CTC in which multiple intermediate compounds were generated with retention times of 2~5 and 7~7.5 min (Figure 4D).

**Figure 5:**
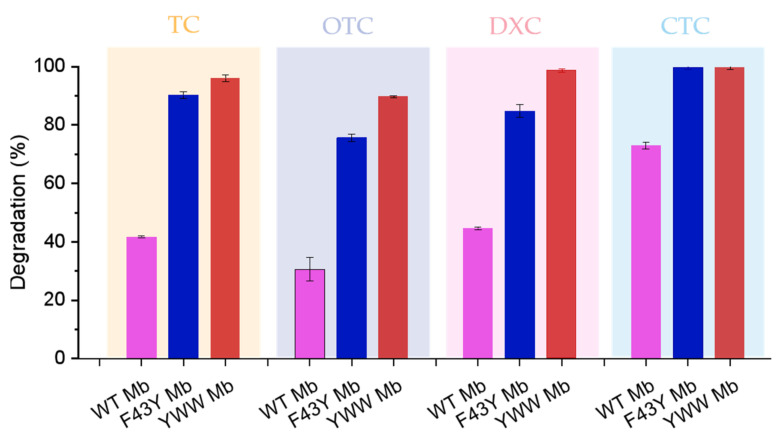
Degradation rates of antibiotics catalyzed by Mb and its mutants. Reaction conditions: 5 μM protein, 400 μM H_2_O_2_, 100 μM antibiotics, 50 mM potassium phosphate buffer at pH 7.0, reaction for 5 min. The error bars represent the standard deviation of the mean.

Based on the HPLC results, we further calculated the degradation rates of the four antibiotics as catalyzed by the Mbs. As shown in Figure 5, YWW Mb and F43Y Mb effectively degraded TC with a degradation rate of ~97% (turnover number, TON, ~19) and ~91% (TON, ~18), respectively, whereas a lower rate of ~41% (TON, ~8) was observed for WT Mb. In the case of DXC degradation, YWW Mb could efficiently degrade DXC with a degradation rate of ~99% (TON, ~20), and F43Y could degrade ~85% (TON, ~17) of DXC. In contrast, the degradation rate of DXC catalyzed by WT Mb was only ~45% (TON, ~9). Remarkably, YWW Mb and F43Y Mb were able to completely degrade CTC, and WT Mb could also degrade CTC by ~75% under the same conditions. Among these tetracycline antibiotics, WT Mb could only degrade ~30% of OTC, meanwhile, YWW Mb and F43Y Mb could degrade ~90% and ~76% of OTC, respectively. In a previous study, Wen and coworkers reported that crude manganese peroxidase (MnP) prepared from Phanerochaete chrysosporium could degrade ~72.5% and ~84.3% of TC and OTC within 4 h, respectively [27]. All the results suggest that YWW Mb is an efficient enzyme and could degrade the tetracycline antibiotics more rapidly and efficiently, as compared with F43Y Mb and WT Mb, as well as other native enzymes such as MnP.

### 2.4. Product Analysis by UPLC-ESI-MS

To reveal the degradation pathways of tetracycline antibiotics catalyzed by YWW Mb, we identified the degradation intermediates based on the UPLC-ESI-MS data (Figure S1, see Supplementary Materials). Considering the structural similarity of tetracycline antibiotics, we speculated that TC, OTC, DXC, and CTC had similar degradation pathways, which mainly include hydroxylation, methyl oxidation, decarbonization, demethylation, alcohol oxidation, etc. As shown in this section, we chose DXC as a representative antibiotic and proposed possible degradation pathways for its degradation catalyzed by YWW Mb.

**Figure 6:**
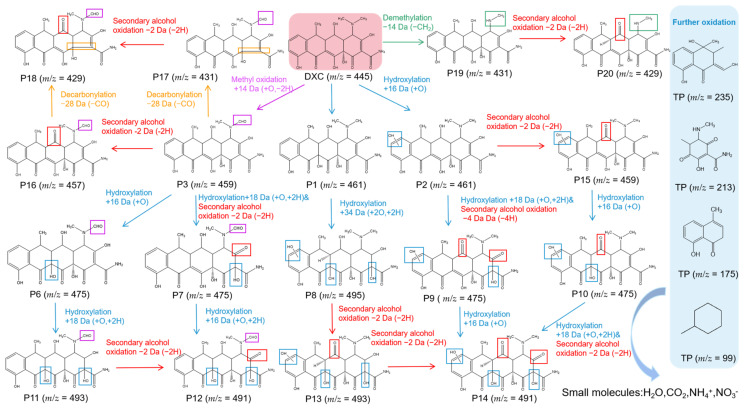
The proposed degradation pathways of DXC as catalyzed by F43Y/F138W/P88W Mb.

As shown in Figure 6, upon the treatment of DXC by YWW Mb, by-products P1 (*m*/*z* = 461) and P2 (*m*/*z* = 461) may be produced by hydroxylation of the C11a = C12 double bond and the aromatic ring D, respectively. Note that the hydroxylation at both sites of P1 will produce the product P8 (*m*/*z* = 495). Similarly, previous studies suggested that the double bonds of C11a = C12 and C2 = C3 on the DXC molecule were prone to be attacked by radicals such as ·OH [28,29,30]. Subsequently, the intermediate products P2 and P8 will generate P13 (*m*/*z* = 493) and P5 (*m*/*z* = 459), respectively, by oxidation of the secondary alcohol. The product P14 (*m*/*z* = 491) will be eventually produced by the secondary alcohol oxidation or hydroxylation of P13, P6, and P5, respectively.

Moreover, DXC will produce P3 (*m*/*z* = 459) by methyl oxidation, which will be decomposed to P15 (*m*/*z* = 431) and P4 (*m*/*z* = 457) by decarbonization and secondary alcohol oxidation, respectively. Both P15 and P4 can further degrade to P16 (*m*/*z* = 429) by decarbonization and secondary alcohol oxidation, respectively. It should be noted that in previous studies, decarbonization and bond cleavage C1-C2 were observed for both ozonation treatment and UV/H_2_O_2_/Fe(II) treatment of DXC [31,32]. Moreover, the product P6 (*m*/*z* = 429) could be formed from P4 by double bond rearrangement of C11a = C12, followed by secondary alcohol oxidation.

In addition, the product P17 (*m*/*z* = 431) might be formed by the demethylation of DXC, likely due to the low energy of the N-C bond, which will further form P18 (*m*/*z* = 429) by the secondary alcohol oxidation [33,34]. These intermediate products will be further degraded into small compounds with small molecular weights of *m*/*z* = 235, *m*/*z* = 213, *m*/*z* = 175, and *m*/*z* = 99, by the cleavage of functional groups, hydroxyl rearrangement, dehydration, etc. With increasing the reaction time, the above intermediates might be ultimately decomposed into even the smallest molecules such as H_2_O, CO_2_, NH_4_^+^, and NO_3_¯.

### 2.5. Toxicity of Tetracycline Antibiotics and Their Degradation Products

**Figure 7:**
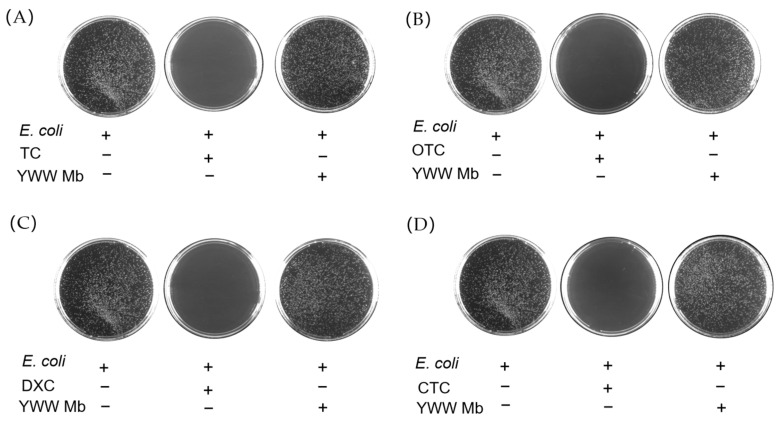
Antibacterial activity of tetracycline antibiotics (**A**), TC; (**B**), OTC; (**C**), DXC; (**D**), CTC, 100 μM) before and after treatment by F43Y/F138W/P88W Mb (5 μM) in the presence of H_2_O_2_ (400 μM). The *E. coli* cells were grown on agar plates at 37 °C for 12 h.

To evaluate the toxicity of TC, OTC, CTC, DXC, and their degradation products, we performed in vitro antibacterial experiments using BL21(DE3) *E. coli* cells. The cell cultures with the addition of different tetracycline antibiotics or their degradation products were incubated on agar plates at 37 °C for 12 h. The toxicity was assessed by the number of colonies of *E. coli* cells. As shown in Figure 7, the colonies on the agar plate without the addition of any antibiotics (blank controls) grew vigorously. Comparatively, in cases of the addition of TC, OTC, DXC, and CTC, there were no *E. coli* cells grown on the agar plates, which indicates that the antibiotics are highly toxic to *E. coli* cells and can completely inhibit their growth. In contrast, when *E. coli* cells were cultured with the degradation products of TC, OTC, CTC, and DXC catalyzed by YWW Mb, the grown colonies were similar to those on the agar plates of blank controls, suggesting essentially negligible toxicity for the degradation products.

Recently, several studies reported that advanced oxidation processes, photolysis, and electro-catalytic treatments of tetracycline antibiotics usually produced highly toxic byproducts [35,36,37]. In contrast, we found that as catalyzed by YWW Mb, the toxicity of TC, OTC, DXC, and CTC degradation products were much lower than that of the parent compounds, similar to those observed for the biodegradation results by bacteria, fungi, and MnP, etc. [38,39,40]. Eventually, the observations indicate that the engineered enzyme YWW Mb is comparable to the native enzyme and is safe for applications in removing antibiotics from the aquatic environment.

## 3. Materials and Methods

### 3.1. Materials

Tetracycline (C_22_H_24_N_2_O_8_·HCl), oxytetracycline (C_22_H_24_N_2_O_9_·HCl), doxycycline (C_22_H_24_N_2_O_8_·HCl), and chlortetracycline (C_22_H_23_ClN_2_O_8_·HCl) were bought from Aladdin Industrial Corporation (Shanghai, China). Wild-type (WT) sperm whale Mb, F43Y Mb, and the triple mutant F43Y/F138W/P88W Mb were expressed and purified as previously reported [41].

### 3.2. UV-Vis Studies

10 mM solutions of TC, CTC, and DXC were prepared by dissolving 19.3 mg, 20.6 mg, and 19.3 mg of each chemical in 4 mL water, respectively. Particularly, a 10 mM solution of OTC was prepared by dissolving 19.8 mg OTC in 3.98 mL water with the addition of 20 μL HCl to a final volume of 4 mL. The assay mixture containing 100 μM substrate, 5 μM YWW Mb, 0.4 mM H_2_O_2_, in 25 mM potassium phosphate buffer (pH 7.0) with a final volume of 2 mL was incubated for 5 min at 25 °C. The absorbance spectra were recorded from 280 to 600 nm on a Lambda 365 spectrophotometer (PerkinElmer, Inc. Waltham, MA, USA). Control experiments were carried out under the same conditions without the addition of H_2_O_2_.

### 3.3. Kinetic Studies

A 10 mM solution of TC (ɛ_254–450nm_ = 1.09 × 10^4^ M^−1^·cm^−1^) [42] was prepared as above mentioned. Then, 100 μM of the substrate, 5 μM YWW, F43Y, or WT Mb, and 0.4 mM H_2_O_2_ were mixed in potassium phosphate buffer (25 mM, pH 7.0) with a final volume of 2 mL. The spectrum was monitored at 357 nm on Agilent 8453 diode array spectrometer (Agilent Technologies, Inc. Santa Clara, CA, USA). Substrate concentrations of TC were varied with a final concentration of 10–100 μM, and the total volume was kept at 2 mL. The time trace of Soret band absorbance was biphasic for the triple mutant (Figure 3A, inset). The corresponding rate constants, *k*_obs1_ and *k*_obs2_, were calculated by fitting to the double-exponential decay function (Equation (1)), and *k*_obs1_ was used for comparison.

*y* = *y*_0_ + *ae*^−*k1t*^ + *ae*^−*k2t*^ (1)

The curves of initial rates versus substrate concentrations (Figure 3B) were fitted to the Michaelis–Menten equation (Equation (2)):

υ/[protein] = *k*_cat_[substrate]/(*K*_m_ + [substrate]) (2)

### 3.4. HPLC Studies

The assay mixture containing 100 μM substrate, 0.4 mM H_2_O_2_, and the 5 μM enzyme (WT Mb or YWW Mb or F43Y Mb) was incubated in 25 mM potassium phosphate buffer (pH 7.0) at 25 °C for 5 min (final volume, 2.0 mL), followed by passing the reaction solutions through a 0.22 mm filter membrane. Then, 20 μL of the filtrate was analyzed by HPLC analysis using a Shim-pack GIST 5 μm C18 column (150/4.6 mm). Elution gradient: 0–10 min, 30% (*v*/*v*) acetonitrile/70% (*v*/*v*) H_2_O/0.1% (*v*/*v*) trifluoroacetic acid. The retention time (RT) of TC, OTC, DXC, and CTC was 6.00, 5.004, 8.121, and 6.890 min, respectively. Eluted substances were detected at 280 nm. Degradation rates of TC, OTC, DXC, and CTC were expressed as a concentration percentage, which was calculated based on the peak area of known standards.

### 3.5. Product Analysis by UPLC-ESI-MS

The enzymatic oxidation products of DXC were monitored by Ultra Performance Liquid Chromatography (UPLC-MS). Sample solutions of DXC were prepared in the same way as previously mentioned. Firstly, 2 mL of reaction mixtures contained 100 μM DXC, 0.4 mM H_2_O_2_, and 5 μM YWW Mb in potassium phosphate buffer (25 mM, pH 7.0). After reaction for 5 min, the reaction solution was passed through a 0.22 mm filter membrane, and then was analyzed in a Waters ACQUITY UPLC/Xevo G2 QTOF system, using a 2.1 mm × 50 mm C18 reverse-phase column (Acquity UPLC BEH, Waters) with a precolumn at 40 °C. The flow rate was set to 0.2 Ml/min, and then the column was further equilibrated with a mobile phase of 70% (*v*/*v*) water (eluent A)/30% (*v*/*v*) acetonitrile with 0.1% (*v*/*v*) trifluoroacetic acid (eluent B) for 5 min. The mass spectrometer was operated in the ESI positive ion modes.

### 3.6. In vitro Antibacterial Experiments

To evaluate the toxicity of TC, OTC DXC, CTC, and their degradation products catalyzed by YWW Mb in the presence of H_2_O_2_ (0.4 mM), BL21(DE3) *E. coli* cells were used as the experimental strain. *E. Coil* cells were cultured in Luria-Bertani (LB) liquid for 12 h on a shaker (220 rpm) at 37 °C, and then 1 μL of *E. Coil* cultures was diluted in 2 mL double-distilled water (dd H_2_O). Experimental groups were set as follows: (1) blank control (*E. coli*, 10 μL); (2) *E. coli* (10 μL) + TC (100 μM, 300 μL); (3) *E. coli* (10 μL) + TC degradation solution (300 μL). Next, all groups were cultured on the agar plates for 12 h at 37 °C for counting the number of colonies. The toxicity of OTC, DXC, CTC, and their degradation products were evaluated using the same methods and procedures.

## 4. Conclusions

In summary, we successfully applied an efficient engineered peroxidase based on Mb, F43Y/P88W/F138W Mb (termed YWW Mb), to catalyze the degradation of tetracycline antibiotics in an aqueous environment. Both UV-vis and kinetic studies showed that the triple mutant YWW Mb could efficiently catalyze the degradation of TC, OTC, DXC, and CTC by using H_2_O_2_ as an oxidant. The degradation products were also analyzed by UPLC-ESI-MS. Based on the results, we proposed the possible catalytic mechanisms for YWW Mb in the degradation of tetracycline antibiotics, which may include multiple degradation pathways, such as hydroxylation, methyl oxidation, decarbonization, demethylation, and secondary alcohol oxidation. In addition, the in vitro antibacterial experiments on *E. coli* cells showed that these tetracycline antibiotics completely lost the antibacterial activity after degradation, indicating non-toxicity for the degradation products. This study reveals that the engineered peroxidase based on Mb with two additional Trp residues is also efficient in the degradation of tetracycline antibiotics, which indicates that the engineered heme enzyme has potential applications in environmental remediation.

## Data Availability

The datasets for this manuscript can be obtained from the corresponding author upon reasonable request.

## Supplementary Materials

The following supporting information can be downloaded at: https://www.mdpi.com/article/10.3390/molecules27248660/s1, Figure S1: UPLC-MS traces of the oxidation products of DXC (100 μM) in the absence (A) and presence (B) of F43Y/P88W/F138W Mb (5 μM) with H_2_O_2_ (0.4 mM) as an oxidant. (C) ESI-MS spectra of DXC. (D–F) Analysis of the oxidation products by ESI-MS spectrometry in a positive mode. Detected products are indicated by orange arrows.

## Sample Availability

The samples of the compounds and/or corresponding spectra are available from the authors.
