# Supplementary material for: Efficient Degradation of Tetracycline Antibiotics by Engineered Myoglobin with High Peroxidase Activity
Source: Molecules. 2022 Dec 7;27(24):8660. doi: 10.3390/molecules27248660 (PMC9782475; doi:10.3390/molecules27248660)
Supplement: Supplementary file 1 [file molecules-27-08660-s001.zip › molecules-2060763-supplementary.pdf]

Supporting Information

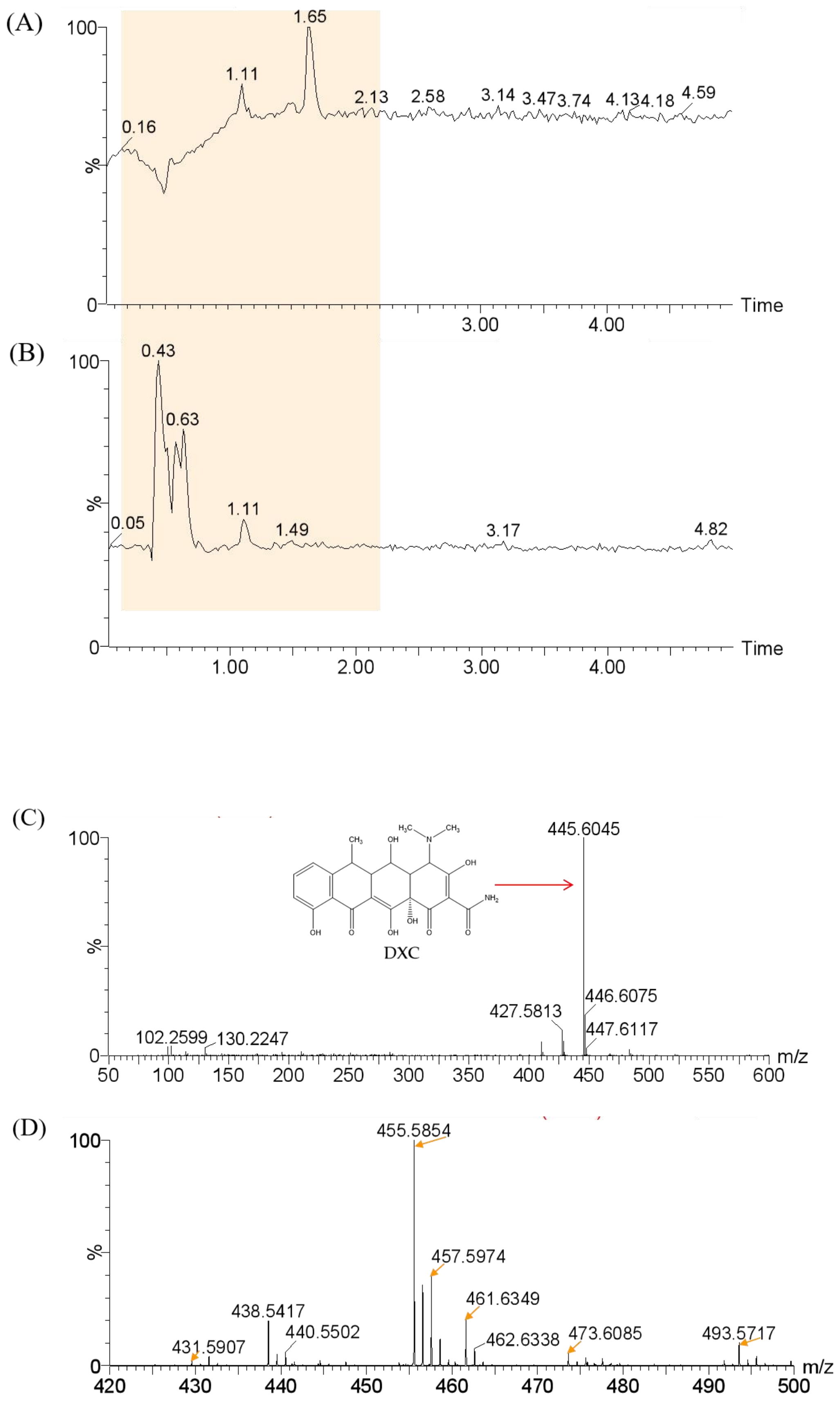

(E)

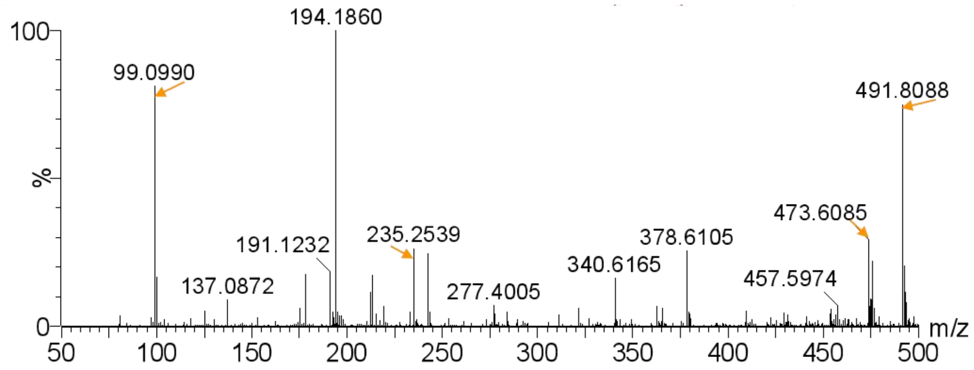

(F)

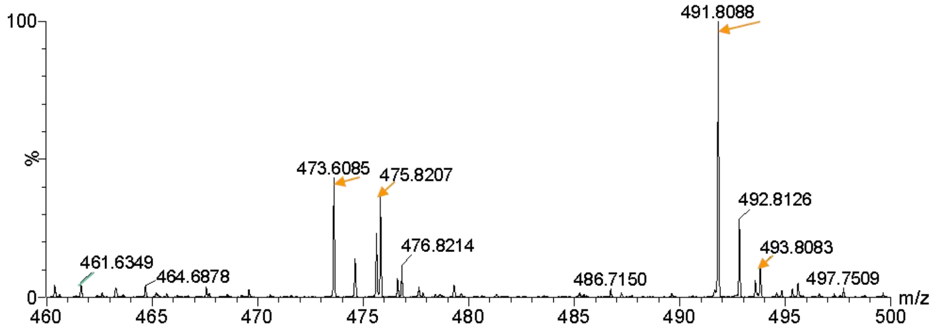

Figure S1. UPLC-MS traces of the oxidation of DXC (100 μM) in the presence (A) and absence (B) of F43Y/P88W/F138W Mb (5 μM) with H_2O_2 (0.4 mM) as an oxidant. (C) ESI-MS spectra of DXC. (D-F) Analysis of the oxidation products by ESI–MS spectrometry in a positive mode. Detected products are indicated by orange arrows.
